# Beyond the one-way ANOVA for ’omics data

**DOI:** 10.1186/s12859-018-2173-7

**Published:** 2018-07-09

**Authors:** Kirsty L. Hassall, Andrew Mead

**Affiliations:** Computational and Analytical Sciences, Rothamsted Research, Harpenden, AL5 2JQ UK

**Keywords:** Multiplicity, Model selection, ’omics, ANOVA

## Abstract

**Background:**

With ever increasing accessibility to high throughput technologies, more complex treatment structures can be assessed in a variety of ’omics applications. This adds an extra layer of complexity to the analysis and interpretation, in particular when inferential univariate methods are applied en masse. It is well-known that mass univariate testing suffers from multiplicity issues and although this has been well documented for simple comparative tests, few approaches have focussed on more complex explanatory structures.

**Results:**

Two frameworks are introduced incorporating corrections for multiplicity whilst maintaining appropriate structure in the explanatory variables. Within this paradigm, a choice has to be made as to whether multiplicity corrections should be applied to the saturated model, putting emphasis on controlling the rate of false positives, or to the predictive model, where emphasis is on model selection. This choice has implications for both the ranking and selection of the response variables identified as differentially expressed. The theoretical difference is demonstrated between the two approaches along with an empirical study of lipid composition in *Arabidopsis* under differing levels of salt stress.

**Conclusions:**

Multiplicity corrections have an inherent weakness when the full explanatory structure is not properly incorporated. Although a unifying ‘single best’ recommendation is not provided, two reasonable alternatives are provided and the applicability of these approaches is discussed for different scenarios where the aims of analysis will differ. The key result is that the point at which multiplicity is incorporated into the analysis will fundamentally change the interpretation of the results, and the choice of approach should therefore be driven by the specific aims of the experiment.

**Electronic supplementary material:**

The online version of this article (10.1186/s12859-018-2173-7) contains supplementary material, which is available to authorized users.

## Background

**Fig. 1:**
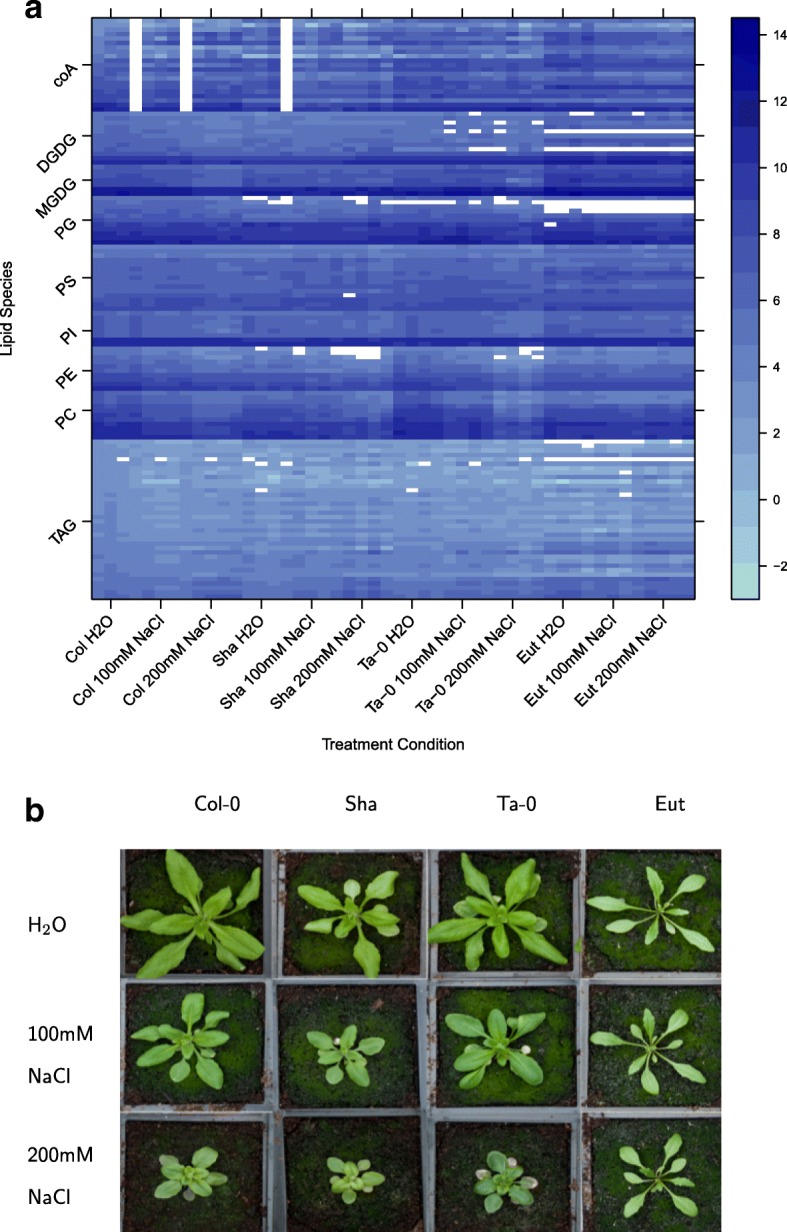
Lipidomics dataset. An experiment to investigate the abundance of 131 lipid species within 4 different Arabidopsis genotypes in 3 differing levels of salt stress. **a** shows a heatmap of the log_2_ abundance of all lipid species in each condition and **b** depicts the factorial design structure

Multivariate data are frequently generated within biological applications due to the ever increasing availability of high-throughput technologies to study functional genomics using transcriptomics, proteomics, lipidomics and metabolomics. These technologies generate data from large numbers of response variables, and typically interest is focussed on identifying the ‘important’ response variables, where important will be a context specific definition. For instance, experiments may be designed with a specific explanatory structure, such as the inclusion of treatment factors, blocking factors or observational covariates, where important variables might be those for which different (combinations of) treatment levels show statistically significant different responses. It is indisputable that this explanatory structure information should be incorporated into the subsequent statistical analysis. However, this is not always straightforward to achieve. This is exemplified in a study of the lipid composition of *Arabidopsis thaliana* under differing treatment combinations, constructed as a replicated 4x3 factorial design for different *Arabidopsis* genotypes (Columbia, Shadara, Ta-0 and Eutrema) under three differing levels of salt stress (0, 100, 200 nM NaCl). In total, the abundance of 131 lipids (response variables) was measured in each condition. The log_2_ abundance over these conditions for the different lipid species is shown in Fig. 1a. Interest might be focussed on lipids showing significant differences between different genotypes, salt stresses or combinations of these.

There are two main avenues of analytical methods that can be applied to such ’omics data; multivariate methods and mass univariate methods (see e.g. [1, 2]). Each have their own advantages and disadvantages, but in combination can elicit a great deal of insight into the underlying mechanisms that generated the data [3–7]. In this paper, we predominantly focus on the issues that arise in the latter methods, the mass-univariate approach, but would like to emphasise that these methods should add to the insight obtained from multivariate approaches rather than act as a replacement.

### Mass univariate analysis

In contrast to multivariate approaches, mass univariate methods investigate each response variable independently. Although the covariance structure between response variables is not accounted for in such an analysis, this approach will provide information about the variability in individual response variables, and, in particular, how it relates to components of the explanatory structure. For example, identifying the sets of treatment conditions that are statistically significant for each response. This is particularly advantageous when an experiment may include complex explanatory structures that include multiple explanatory terms. Standard statistical techniques such as ANOVA (analysis of variance), REML (restricted maximum likelihood) and regression can be used to investigate both the biological size and statistical significance of different explanatory model components in an inferential framework. Moreover, these analysis approaches can cope with complex treatment structures (such as the factorial treatment structure in the above example), unbalanced design structures and missing values.

In generality, it is of interest to fit and analyse the following linear model,

1 $$\begin{array}{*{20}l}  y_{i} &= X_{i} \beta + \epsilon_{i}, \; \text{for}\ i=1,...,n \\ \epsilon_{i} &\sim N(0, \sigma^{2}),  \end{array} $$

where *n* is the number of observations, *y*_*i*_ is the *i*th observation of a single response variable **y**, *X*_*i*_ is the *i*th row of the design matrix **X**, *β* is the vector of coefficients to be estimated and *ε* are independent error terms. Fitting and analysing such models is a completely standard statistical approach using the methods identified above.

However, there are issues in applying such methods en masse to many different response variables in the same dataset. To illustrate this, let us first consider the simple example where the design matrix **X** corresponds to a single explanatory factor. Specifically, suppose

2 $$\begin{array}{*{20}l} y_{ji} &= \beta_{j} + \epsilon_{ji}, \; i=1,...,n_{j}, \; j=1,...,J  \end{array} $$

where *y*_*ji*_ is the *i*th observation on the *j*th treatment. Once fitted, the estimated parameters of this model can be assessed. Specifically, using either a two-sample t-test (for *J*=2) or an F-test (for *J*>2) the null hypothesis of no difference in response in the *J* different treatments can be tested. When testing at a pre-specified significance level, *α*, of 0.05, the probability of a type I error (the false positive rate) is controlled at 5%. However, it is well known that applying such a univariate analysis approach, en masse, to multiple response variables, will result in an unacceptably high type I error rate over the whole experiment due to the issue of multiple testing (also referred to as multiplicity), described below.

#### Multiple testing

In a multiple testing scenario, the probability of a type I error, or false positive (i.e. the probability of rejecting the null hypothesis when the null hypothesis is true) over the tests for the complete set of response variables is inflated. For example, consider making independent hypothesis tests for 10 (independent) response variables, the probability of making at least one type I error, *α*^∗^, is given by,

$$\begin{array}{*{20}l} \alpha^{*} & = \text{Prob}(\text{at least 1 type I error}) \\ & = 1- \text{Prob}(\text{no type I error}) \\ &= 1 - (1 - \alpha)^{10}, \end{array} $$

which when the individual level of significance is *α*=0.05, gives *α*^∗^=0.40. Thus, to control the overall probability over *R* tests (the number of response variables) of at least one type I error at a specified level *α*, the significance level for each test becomes 1−(1−*α*)^1/*R*^, which in practice is often approximated by the upper bound *α*/*R*, the Bonferroni correction. This adjustment controls the family wise error rate (FWER), defined to be the probability of at least one type I error. Alternative ways of controlling the type I error in a multiplicity framework include controlling the per-family error rate (PFER), which is defined to be the expected number of type I errors, and the false discovery rate (FDR), defined as the expected proportion of false discoveries. A plethora of methods for controlling the type I error, through differing multiple testing corrections, can be found in the literature (see [8, 9] and [10] for reviews). These often have different aims, such as which error rate is to be controlled, whether or not tests can be considered independent and whether the chosen error rate should be controlled in the strong or weak sense [8].

It is reasonable in an ’omics framework to assume there will be substantial positive dependence between response variables. This means that any correction based on the assumption of independent tests will be highly conservative and will consequently reduce the power of the test for fixed sample sizes. Alternative procedures are available that account for this dependence, for example [11] employed resampling methods and [12–14] adjusted the significance level based on an effective number of independent tests *N*_eff_<*N*.

Where there is only a single explanatory variable in the model, as in Eq. 2, any of the above multiplicity corrections can be incorporated without difficulty to ensure the overall type I error rate of the whole experiment does not become over-inflated.

#### Incorporating explanatory structures

However, there is a lack of coherence within the literature when explanatory structures become more complex. As the cost of ’omics experiments decreases, experimenters are increasingly generating ’omics datasets from experiments with more complex designs, including both crossed and nested treatment structures. Examples can be found in transcriptomics [15–17], proteomics [18, 19] and metabolomics (as exemplified from the open source database PMR [20]). In all of these scenarios, mass univariate analysis can be applied to investigate the effect of the explanatory structure on a response-by-response basis.

Consider, the salt stress lipidomics example. For a single response variable, the associated linear model takes the form,

3 $$ {}\begin{aligned} y_{jki} &= \mu \,+\, a_{j} + b_{k} + (ab)_{jk} \,+\, \epsilon_{jki}, \; \text{for}\ j=1,...,4; k=1,...,3 \\ \epsilon_{jki} &\sim N(0, \sigma^{2}), \end{aligned}  $$

where *y*_*jki*_ is the response due to the *i*th observation on the *j*th genotype for the *k*th salt stress, *a*_*j*_ is the effect associated with genotype *j*, *b*_*k*_ is the effect associated with salt level *k*, (*ab*)_*jk*_ is the interaction effect of genotype *j* and salt level *k* and *ε* are independent error terms.

For a single response variable (e.g. lipid TAG 52.6), the corresponding analysis would be done using ANOVA to produce Table 1. This analysis provides an assessment of the statistical significance of the three individual terms, through independent tests, according to the explanatory model given in Eq. 3. The question then arises as to how to apply the multiplicity correction when applying such an analysis to multiple response variables. All developed multiplicity correction approaches are only appropriate for correcting a single hypothesis test per response variable as in the example corresponding to Eq. 2. Thus to directly apply these multiplicity corrections, the complex treatment structure would have to be ignored and the multiplicity correction instead applied to the one-way unstructured analysis. For the salt stress lipidomics example, this corresponds to the analysis of the one-way (unstructured) treatment term in the following linear model:

4 $$\begin{array}{*{20}l}  y_{jki} & = \mu_{jk} + \epsilon_{jki}, \; \text{for}\,\, j=1,...,4; k=1,...,3 \\ \epsilon_{jki} &\sim N(0, \sigma^{2}),  \end{array} $$

**Table 1:** The two-way ANOVA table for a single lipid response variable (TAG 52.6) with treatment factors genotype and salt

	Df	Sum Sq	Mean Sq	F value	Pr(>F)
Genotype	3	27.503	9.168	38.230	< 0.001
Salt	2	11.852	5.926	24.711	< 0.001
Genotype:Salt	6	2.328	0.388	1.618	0.171
Residual	36	8.633	0.240		

where *μ*_*jk*_ is the mean response for genotype *j* and salt level *k*. The associated ANOVA table is given in Table 2. This analysis clearly fails to exploit the factorial treatment structure included in the design of the experiment, providing no information about the relative sizes and importance of the two main effects and the interaction effect.

**Table 2:** The corresponding one-way unstructured ANOVA table for a single lipid response variable (TAG 52.6), where Genotype:Salt is a single explanatory term corresponding to all combinations of genotype and salt conditions

	Df	Sum Sq	Mean Sq	F value	Pr(>F)
Genotype:Salt	11	41.682	3.789	15.802	< 0.001
Residual	36	8.633	0.240		

In recent years, developments in two-stage or hierarchical hypothesis testing [21] have gone some way to addressing these issues. In this framework, a family of hypotheses and tests are associated with each response variable. In the first stage an overall test of the combined effects of all explanatory variables is considered, with more detailed, individual hypotheses of interest only considered where this first overall test gives a significant result. In this way the overall false discovery rate (OFDR) [22] can be controlled across all response variables. Examples using such an approach include the analysis of data from microarray gene expression studies with multiple treatments [23] where at the first stage the OFDR is controlled for the overall F-test resulting from a one-way ANOVA for each gene. Subsequently, at the second stage of testing, ad-hoc pairwise comparisons of interest between treatments are considered only for genes having a multiplicity-corrected significant result for the overall test (potentially requiring further corrections for multiple comparisons). Two similar approaches (referred to as a nested F and a hierarchical F method) are implemented in the decideTests function of the limma package [24] for differential expression analysis of microarray and RNAseq data. Heller et al. [25] also considered a two-stage approach for the analysis of data from microarray gene expression studies, but here the first stage considered the overall analysis for a gene set, with the OFDR controlled at the gene set level, and the second stage then assessing individual genes within those gene sets initially identified as showing significant results. More recently, Van den Berge et al. [26] extend this concept to RNAseq data to account for factorial treatment structures. Although these approaches enable multiplicity to be controlled at the appropriate level, i.e. at the level of the gene or set of genes, or as in our case at the response level, rather than at the hypothesis level, the second stage of testing is not always fully satisfactory. The examples above all focus the second stage testing on pairwise comparisons requiring a second multiplicity control of the FWER and therefore a reduced power compared to that achieved when fully exploiting the explanatory structure. We extend these approaches by exploiting the full structure of the explanatory model both at the first and second stage of testing. Moreover, we focus on developing visualisations from the output of such approaches in order to explicitly investigate the structure of the data. Explicitly, we aim to provide mass univariate analysis approaches that can both:

- identify which response variables show significant levels of variation due to the explanatory model (taking account of multiplicity), and
- assess how these response variables are important by identifying the components of the explanatory model showing significant effects.

In the following, we present a general paradigm of analysis, through which we develop the theory behind two methods for incorporating a multiplicity correction within the general linear model framework. This is supported by a simulation study before demonstrating the methods on an application in lipidomics, where we investigate the biological understanding that can be gained from such an approach. We end with a discussion on the methods, highlighting current limitations and possible extensions.

## Methods

Typically, linear models as defined in (1) are used both to estimate the effects due to individual explanatory variables (or the interactions between two or more explanatory variables), and to test the statistical significance of these effects. For the analysis of a designed experiment, it is conventional to fit the saturated (or maximal) model, and it is important to make a distinction between this model and the predictive model, used to generate predictions for different combinations of the explanatory variables (usually factors). The saturated model includes all of the explanatory terms associated with the factors included in the design of the experiment, and provides the basis for assessing the statistical significance of each of these terms (blocking factors, main effects of treatment factors and interaction effects between two or more treatment factors). Having determined the statistically significant effects relative to the estimated background (observation-to-observation) variability, the predictive model can be formed, containing all statistically significant terms plus terms that are marginal to these (i.e. both main effects must be included in the predictive model if the associated interaction effect is statistically significant). Where an experiment is carefully designed to be balanced and orthogonal, this selection of the predictive model is straightforward as all terms in the saturated model will be independent. For more general explanatory structures, it may be more difficult to define a saturated model, so that the fitted model will usually be the predictive model.

In both scenarios, the identification of the terms in the predictive model can be achieved in multiple ways; such as using F-tests to assess individual explanatory terms, likelihood ratio tests to compare nested models, or the minimisation of an information criterion. In some cases these approaches will be equivalent. Note that, with the exception of model selection via information criteria, all methods rely on a predefined level of statistical significance at which to perform the test used to include or omit a term from the predictive model. In these scenarios, selection of the predictive model will identify the statistically significant explanatory terms for each response variable. Thus, we consider the incorporation of multiplicity correction and the selection of a response-specific predictive model in a number of different ways. The process for achieving this can be split into three steps,

- A ranking of responses in order of significance [RANK]
- A filtering process to discard non-significant responses (incorporating corrections for multiplicity) [FILTER]
- A model selection step to define the predictive model (i.e. to choose the important terms in the explanatory structure) for each response [MODEL].

The order in which these steps are implemented will depend upon the aims of the study.

### Approach 1: rank, filter, model (RFM)

Firstly, we consider the RFM approach, where responses are ranked, filtered and then modelled. Explicitly, this approach can be viewed as an extension to the one-way multiplicity corrections of Eq. 4, and consists of the following steps:

1. For each of the *n* response variables, fit the linear model with a single one-way (unstructured) treatment term and calculate the associated one-way ANOVA as in example (4) to obtain an overall test of significance at this first step.
2. Rank the responses based on the significance of this overall test and apply a multiplicity correction of choice to this set of *n* tests.
3. Filter out the responses deemed non-significant after the multiplicity correction.
4. For the remaining response variables, apply a model selection process to the full explanatory structure, generating a predictive model for each significant response variable.

**Fig. 2:**
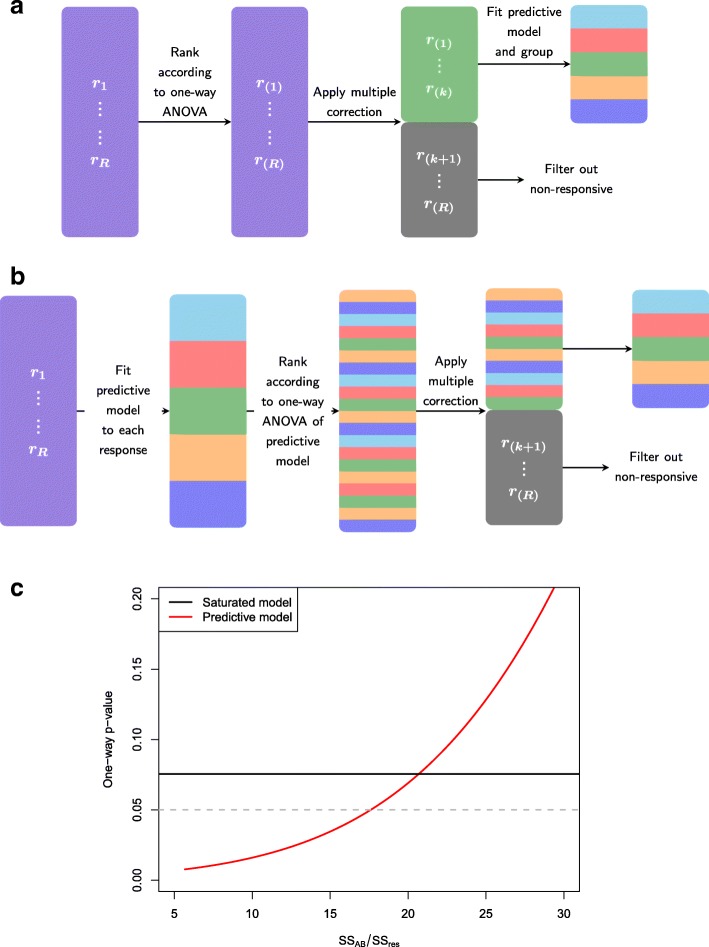
Pictorially representation of two approaches to incorporate multiplicity into non-trivial explanatory structures. **a** Option 1. Rank, filter, model (RFM). This approach first applies a multiplicity correction to the one-way ANOVA obtained from the saturated model to filter out non-significant responses and then applies a model selection step to obtain a response specific predictive model. **b** Option 2, Model, rank, filter (MRF). This approach first performs a model selection step to obtain a response specific predictive model, from which a one-way ANOVA is applied and corrected for multiplicity to filter out non-significant responses. **c** For the two-factor model in Eq. 5 with a 3x4 factorial treatment structure and a replication of 3, this shows the relationship between the one-way ANOVA obtained from a saturated model (fixed variance ratio of 2 and associated significance value of 0.075 – shown by the solid black line) and the one-way ANOVA obtained from the predictive model (for an increasing interaction effect). Thus, as the variance ratio of the interaction term (shown by the solid red line) decreases, the associated *p*-value of the one-way ANOVA of the predictive model also decreases

This process is depicted in Fig. 2a. Since there are a finite number of possible models, responses can be grouped according to the set of significant terms in the predictive model. In the case that model selection is done via information criteria, this is a simple extension to current methods, which enables the underlying explanatory structure in each response to be further investigated. If however, model selection is applied via a testing procedure, such as likelihood ratio tests or F-tests, then careful consideration of the chosen level of significance is required. Specifically, in order to make consistent comparisons, the significance level for the model selection step for each response should be adjusted according to the multiplicity correction in step 2.

To be explicit, let *p*^(1)^,...,*p*^(*R*)^ be the unadjusted *p*-values for the responses 1...*R* of the one-way F-test in step 1. After a multiplicity correction in step 2, the adjusted *p*-values are given by max(1,*c*^(1)^*p*^(1)^),..., max(1,*c*^(*R*)^*p*^(*R*)^), where *c*^(*r*)^ is the adjustment applied to the *p*-value for response *r* (e.g. under a Bonferroni correction *c*^(*r*)^=*R*, ∀*r*∈{1...*R*}). Equivalently, the significance level, the probability of a type I error, has also been adjusted by 1/*c*^(*r*)^, which under a Bonferroni correction is 1/*R*. Thus, each response is assessed against a specific significance level *α*/*c*^(*r*)^, *r*=1...*R*. This response specific significance level is carried through to step 3 and is the significance level used in the model selection step.

### Approach 2: model, rank, filter (MRF)

An alternative approach is the MRF approach, which starts with the model selection step and filters the response variables based on an overall test of the predictive model. Explicitly, this consists of the following steps,

1. For each response variable, apply a model selection process to the full explanatory structure. This will then define specific explanatory structures for each response, yielding the predictive model for each of the *n* responses. Note, no multiplicity correction is applied at this step as the aim is to construct a predictive model for each response based on a consistent measure of significance.
2. For each response-specific predictive model, obtain the associated one-way (unstructured) treatment term and fit the corresponding linear model that has a single treatment term to obtain an overall test of significance through a one-way ANOVA.
3. Rank the responses based on the significance of this overall test and apply a multiplicity correction of choice to this set of *n* tests.
4. Filter out the response variables deemed non-significant after the multiplicity correction.

This is depicted in Fig. 2b. As with RFM, this will result in different responses having different significant explanatory variables and hence can be used to classify responses according to their final explanatory structure. However, in contrast to RFM, the model selection step in MRF, regardless of which method used, will be consistent across all response variables, whilst the overall test for significance will differ. Since this test is applied after model selection, models for different response variables will have a differing number of explanatory terms and as such the overall F-test will have response-specific numerator and denominator degrees of freedom.

### A comparison

Although the general framework is very similar for incorporating multiplicity corrections and model selection in these two approaches, they give fundamentally different classifications of the response variables. The approach that should be used will depend on the scenario in question. In the case of balanced orthogonal designs, where all model terms are either blocking or treatment factors, the RFM approach seems favourable as the multiplicity correction is applied to the saturated model. In a regression framework, where model terms may include observational variables, intuitively model selection is more important, motivating the use of MRF which puts more emphasis on the model selection process.

To illustrate where differences occur between the two approaches, consider the example of a designed experiment consisting of a two factor (A and B) *t*_A_ x *t*_B_ factorial treatment structure (with no blocking structure). The saturated model is given by,

5 $$\begin{array}{*{20}l} y_{jki} = a_{j} + b_{k} + (ab)_{jk} + \epsilon_{jki},  \end{array} $$

with corresponding ANOVA table given in Table 3.

**Table 3:** A general two-way ANOVA table consisting of a factorial treatment structure between factors A and B

	df	SS	MS	F
A	(*t*_A_−1)	*S* *S*_A_	$\tfrac {1}{(t_{\mathrm {A}} - 1)} {SS}_{\mathrm {A}}$	*MS*_A_/*MS*_res_
B	(*t*_B_−1)	*S* *S*_B_	$\tfrac {1}{(t_{\mathrm {B}} - 1)}{SS}_{\mathrm {B}}$	*MS*_B_/*MS*_res_
A:B	(*t*_A_−1)(*t*_B_−1)	*S* *S*_AB_	$\tfrac {1}{(t_{\mathrm {A}} - 1)(t_{\mathrm {B}} - 1)}{SS}_{\text {AB}}$	*MS*_AB_/*MS*_res_
Residual	*N*−*t*_A_*t*_B_	*S* *S*_res_	$\tfrac {1}{(N-t_{\mathrm {A}}t_{\mathrm {B}})}{SS}_{\text {res}}$	

Under RFM, the overall significance test is based on the one-way ANOVA and is given by the test statistic,

$$\begin{array}{*{20}l} \mathrm{F}_{A*B} & = \frac{({SS}_{\mathrm{A}} + {SS}_{\mathrm{B}} + {SS}_{\text{AB}})/(t_{\mathrm{A}}t_{\mathrm{B}} - 1)}{SS_{\text{res}}/(N-t_{\mathrm{A}}t_{\mathrm{B}})} \\ & = \frac{N-t_{\mathrm{A}}t_{\mathrm{B}}}{t_{\mathrm{A}}t_{\mathrm{B}} - 1} \times \left(\frac{SS_{\mathrm{A}}}{SS_{\text{res}}} + \frac{SS_{\mathrm{B}}}{SS_{\text{res}}} + \frac{SS_{\text{AB}}}{SS_{\text{res}}} \right), \end{array} $$

where under the null hypothesis that there is no difference between the *t*_A_×*t*_B_ different treatments, $\phantom {\dot {i}\!}\mathrm {F}_{A*B} \sim F_{t_{\mathrm {A}}t_{\mathrm {B}} - 1, N-t_{\mathrm {A}}t_{\mathrm {B}}}$.

Now, let us assume that the interaction term in the two-way structured ANOVA is not significant, i.e. the F statistic given by,

$$\begin{array}{*{20}l} \mathrm{F}_{A:B} & = \frac{SS_{\text{AB}}/((t_{\mathrm{A}}-1)(t_{\mathrm{B}} - 1))}{SS_{\text{res}}/(N-t_{\mathrm{A}}t_{\mathrm{B}})} \\ & = \frac{N-t_{\mathrm{A}}t_{\mathrm{B}}}{(t_{\mathrm{A}}-1)(t_{\mathrm{B}} - 1)} \times \left(\frac{SS_{\text{AB}}}{SS_{\text{res}}} \right), \end{array} $$

is less than the 5% critical value ($\mathrm {F}_{A:B} < F^{[0.05]}_{(t_{\mathrm {A}}-1)(t_{\mathrm {B}} - 1), N-t_{\mathrm {A}}t_{\mathrm {B}}}$). Then under the MRF procedure, the interaction term is dropped from the predictive model and the overall assessment of significance is based on the test statistic,

$$\begin{array}{*{20}l} {}\mathrm{F}_{A+B} = \frac{N-(t_{\mathrm{A}}+ t_{\mathrm{B}})}{t_{\mathrm{A}} + t_{\mathrm{B}} - 1} \!\times\! \left(\frac{SS_{\mathrm{A}}}{SS_{\text{res}} + {SS}_{\text{AB}}} \,+\, \frac{SS_{\mathrm{B}}}{SS_{\text{res}} + {SS}_{\text{AB}}} \right), \end{array} $$

on *t*_A_+*t*_B_−1 and *N*−(*t*_A_+*t*_B_) degrees of freedom.

Thus, the overall test statistic under RFM (given by F_*A*∗*B*_) can be directly related to the overall test statistic under MRF (given by F_*A*+*B*_), see Section 1 of the Supplementary Material in Additional file 1 for details. This relationship is shown in Fig. 2c for *t*_A_=4,*t*_B_=3, *N*=36 and overall F-statistic of F_*A*∗*B*_=2, which implies an associated *p*-value of *p*_*A*∗*B*_=0.075. Thus, regardless of the significance of any individual main effects, under RFM this response variable would be filtered out as being non-significant. However, as can be seen in Fig. 2c, when the interaction term is sufficiently non-significant (and consequently one or more main effects are highly significant since the overall variance ratio is fixed at 2), MRF would correctly identify a significant response variable. Thus, RFM is a conservative approach that has the potential for prematurely filtering out responses.

By moving the filtering step of the RFM approach to post-model selection step (i.e. a rank model filter (RMF) approach as described in Additional file 1 of the Supplementary Material), this conservativeness can be mitigated, but ranking in the first step will, in general, result in a more conservative multiplicity correction compared to the MRF approach that applies multiplicity corrections to the one-way test of the predictive model associated with larger residual degrees of freedom.

## Results

The methods derived above are demonstrated and compared through a comprehensive simulation study before being applied to the lipidomics dataset previously described. For this dataset, we also consider in detail the interpretation of such analyses through the presentation and visualisation of the output.

### Simulation study

In order to comprehensively compare these methods, our simulation study considered three different designs (a 2×2 factorial treatment structure, a 3×2×4 factorial treatment structure and a 3×2×4 factorial treatment structure with an imposed randomized complete block structure). For each design, we varied the number of responses (500, 1000 and 20000), the number of replicates (3, 4 and 5) and the multiplicity control (FDR via Benjamini-Hochberg and FWER via Bonferroni). In addition, for the randomized complete block design responses were simulated with a block diagonal dependence structure. Each scenario was simulated 50 times in order to obtain representative estimates of the achieved error rates under the two methods of interest (RFM and MRF). Thus, in total, 1350 datasets were simulated covering a range of design scenarios, each of which was analysed via the following four methods.

1. RFM, with Benjamini-Hochberg (B-H) multiplicity correction controlling the FDR at 5% and model selection via F-tests
2. MRF, with Benjamini-Hochberg multiplicity correction controlling the FDR at 5% and model selection via F-tests
3. RFM, with Bonferroni multiplicity correction controlling the FWER at 5% and model selection via F-tests
4. MRF, with Bonferroni multiplicity correction controlling the FWER at 5% and model selection via F-tests

To compare the RFM and MRF approaches, we investigated the observed error rates within the simulation study. Throughout, we focus our discussion to the analysis under a B-H multiplicity correction. Further discussion under a Bonferroni multiplicity correction is given in Section 3.3 of the Supplementary Material in Additional file 1.

**Fig. 3:**
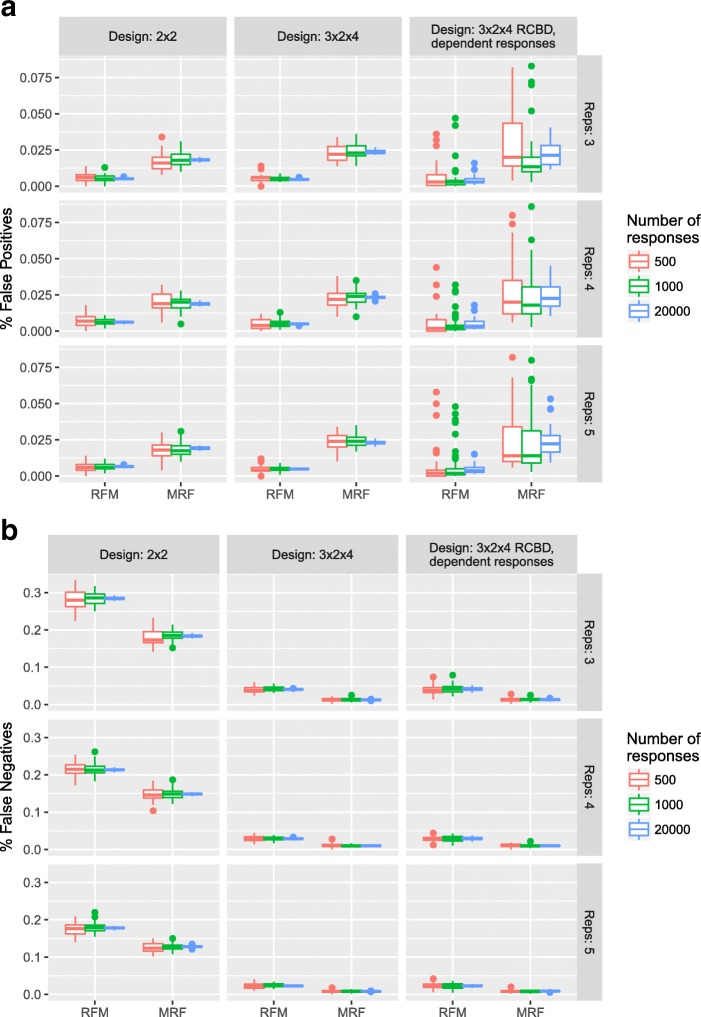
Simulation Study: Overall Type I and Type II error. Under a B-H control of the FDR: **a** Boxplots showing the distribution of the percentage of responses (within a dataset) falsely identified as having non-constant mean response and **b** Boxplots showing the distribution of the percentage of responses (within a dataset) falsely identified as having a constant mean response over all treatment groups

Figure 3a shows the distribution of the percentage of false positives (out of a total of *R* responses) for each simulation scenario. From this, it can be seen that under the MRF, more false positives are detected than under the RFM. However, both methods appear to control the FDR (as shown empirically in Section 3.2 of the Supplementary Material in Additional file 1). As the number of responses increases, the distribution of error rates tightens with little difference observed as the sample size increases. Moreover, it is clear that where there is a strong dependence structure between responses there is a greater variability in the type I error rate obtained under the MRF. Since model selection is emphasised under the MRF, this approach would likely benefit by taking explicit account of the dependence structure in the modelling step, for example through a hierarchical approach. The distribution of the percentage of false negatives (the number of responses falsely identified as non-significant out of a total of *R* responses) is shown in Fig. 3b. Under the MRF, fewer false negatives are detected than under the RFM as predicted from Fig. 2c. Moreover, it can clearly be seen that as the power of the tests increases for larger sample sizes and more complex experiments, the rate of false negatives decreases.

**Fig. 4:**
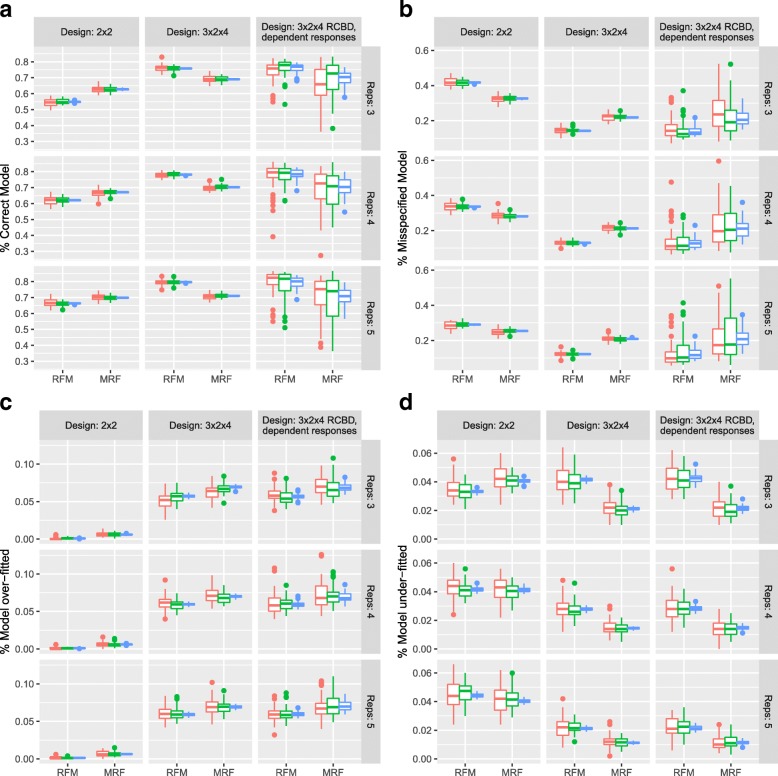
Simulation Study: Model misspecification. Under a B-H control of the FDR: **a** Percentage of responses with the correct model specification identified from each of the RFM and MRF approaches compared to the true generating process. **b** Percentage of responses with a model misspecification identified from each of the RFM and MRF approaches compared to the true generating process. **c** Percentage of responses that have been over-fitted (the fitted model includes all terms from the true generating process with additional terms) under the RFM and MRF approaches. **d** Percentage of responses that have been under-fitted (the fitted model includes only a subset of terms, and no others, from the true generating process) under the RFM and MRF. A model misspecification is defined to be a model that differs from the true generating process in a way not captured by over- or under-fitting. Red, green and blue correspond to simulations of 500, 1000 and 20000 responses respectively

However, the identification (or mis-identification) of overall significance is not the only error we can consider. Figure 4 shows the rate of model misspecification under the RFM and MRF approaches. There is a tendency for the MRF to have a higher rate of correct model specification in the 2×2 design structure compared to the RFM. Moreover, the MRF appears slightly worse under a 3×2×4 structure, although this difference is diminished under a Bonferroni multiplicity correction (Section 3.3 of the Supplementary Material in Additional file 1). Both methods have a higher rate of over-fitting compared to the rate of under-fitting for the more complex design structure (Fig. 4c and d). Moreover, in these more complex design scenarios, the RFM generally has a higher rate of under-fitting than the MRF (Fig. 4d) providing a more parsimonious representation of the response but at the cost of a conservative filtering of overall differential expression.

### Case study: Lipidomics

Applying both RFM and MRF under a B-H correction to control the FDR to the lipidomics dataset described previously, only minor differences in the number of lipids identified as showing differential expression can be seen. Specifically, of the 131 lipids, 129 were identified as showing differential expression between treatments under RFM, whilst under MRF all 131 lipids were identified. However, discrepancies between the methods become apparent when assessing the selected predictive model, with around 4% fewer lipids found to have a significant interaction term under RFM compared with MRF. These differences were exacerbated with the application of a more stringent Bonferroni correction (not shown).

Although the identification of ‘significant’ response variables is useful, greater insight can always be gained by coupling the notion of statistical significance to biological relevance. Within the ’omics framework, it is common to use high throughput technologies to identify a list of the most ‘interesting’ response variables to investigate further. In the simplest case, where comparisons are only made between two treatment conditions, comparisons are often visualised through volcano plots, which show the relationship between statistical significance (often scaled logarithmically) and biological relevance (often presented in terms of fold change to a baseline condition). This enables easy identification of the ‘most interesting’ responses that are both statistically significant and biologically relevant. However, for more complex explanatory (treatment) structures, it is far from clear how to identify ‘interesting’ responses.

#### Overall assessment

**Fig. 5:**
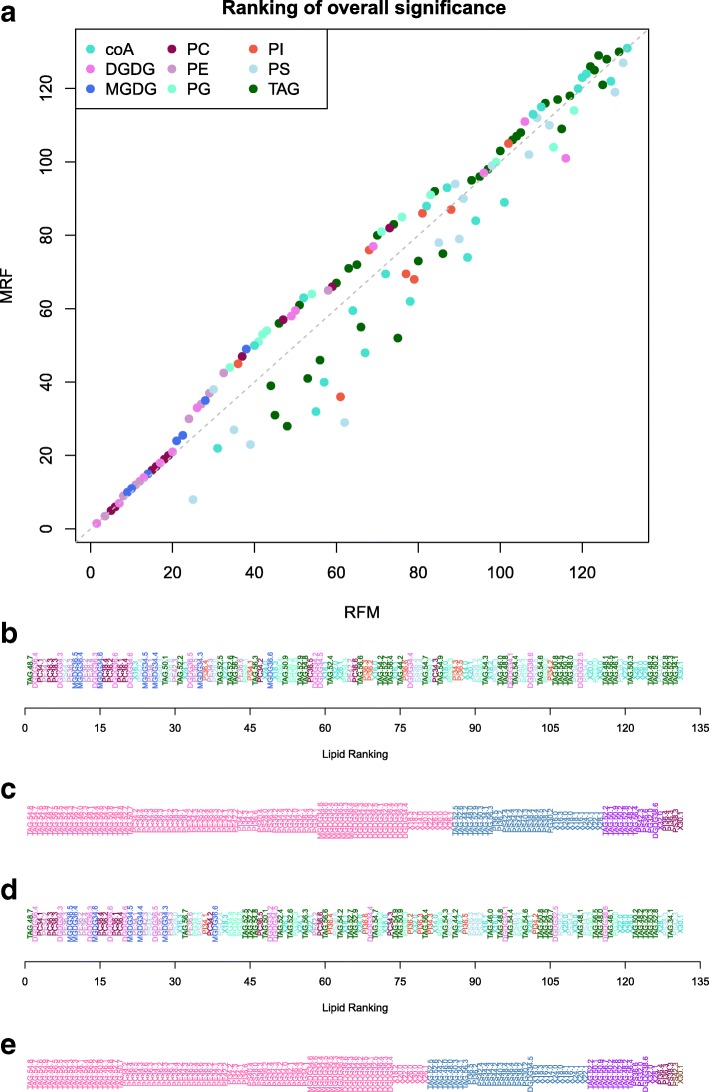
Lipidomics Case Study: Overall Comparison. **a** Comparison of the ranking of the set of lipid species obtained from the overall measure of statistical significance via the RFM approach compared to the MRF approach. Lipids above the grey dashed line are ranked higher under RFM, whereas lipids below the dashed line are ranked higher under MRF. Colour of points indicates the associated type of each lipid species. **b** Lipids ranked according to the overall test of significance under MRF (one-way ANOVA of the predictive model). **c** Lipids grouped according to the set of effects included in the predictive model under MRF. **d** Lipids ranked according to the overall test of significance under RFM (one-way ANOVA of the saturated model). **e** Lipids grouped according to the set of effects included in the predictive model under RFM. Information provided in tabular form in Additional file 2 of the Supplementary Material

In both the RFM and MRF approaches, a one-way F-test is applied to obtain an overall level of significance. The meaning of this one-way test is fundamentally different in the two approaches since under RFM, it is a test of the saturated model, whereas under MRF, it is a test of the predictive model. Nevertheless, this measure of statistical significance can be used to rank responses. Figure 5 shows the discrepancy in the ranking of responses from the two methods, with RFM giving a greater rank to response variables with significant interaction components, whilst MRF gives greater rank to responses with parsimonious predictive models (relative to the corresponding saturated model).

In addition, these approaches can be used to characterise the response variables through the identified predictive model. For example, the lipidomics response variables can be categorised into 8 different groups, where each group contains response variables with the same predictive model. As shown in the simulation study, the predictive models identified by the two approaches differ and so different categorisations of the response variables are obtained as shown in Fig. 5c and e.

More meaningful insight can be obtained by coupling these overall measures of significance with measures of biological relevance. One approach to obtaining measures of biological relevance is through predictions obtained from the predictive model selected in the two-step analysis procedures. Since an overall assessment of significance based on the saturated model (as under RFM) may have very little relevance to predictions from the predictive model, the overall assessment of significance based on the predictive model (as under MRF) should be used.

**Fig. 6:**
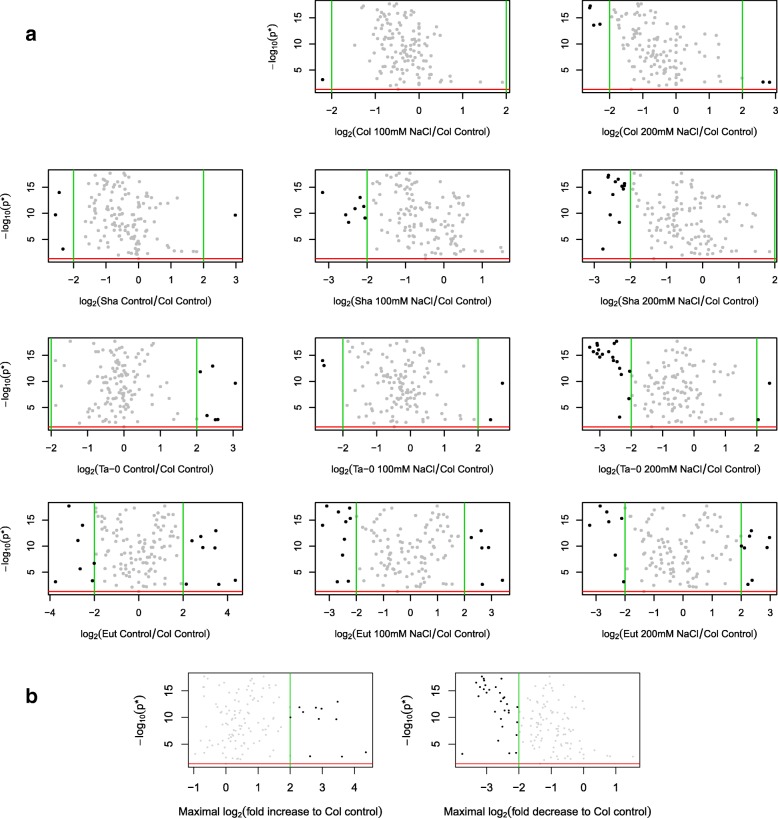
Lipidomics Case Study: Overall Assessment under the MRF. **a** Coupling the overall measure of statistical significance (of the one-way test under MRF after a Benjamini-Hochberg multiplicity correction) with measures of biological relevance calculated as the predicted fold change in abundance compared to the baseline treatment of no salt stress for the variety Columbia (Col). **b** Coupling the overall measure of statistical significance (of the one-way test under MRF after a Benjamini-Hochberg multiplicity correction) with a generalised measured of biological relevance calculated as the maximal fold increase (or decrease) under any treatment condition compared to the baseline treatment of no salt stress for the variety Columbia (Col). Red lines indicate a *p*-value of 0.05. Green lines indicate a fold change of 4

Model predictions are a useful way of defining biological relevance, for example through the predicted fold change between two treatment conditions. However, when there are more than two treatment conditions multiple measures of biological relevance can be obtained and the most meaningful comparisons will be context specific. For example, the treatment condition of the *Arabidopsis* variety Columbia under no salt stress can be considered as a baseline or default status. In this scenario, a measure of biological relevance can be obtained as the predicted fold change (in each treatment condition) from the baseline level. This results in a series of pairwise comparisons to a baseline and can be represented through a *set* of volcano plots (Fig. 6a) where the measure of significance is constant across all plots, but with each subplot reflecting the fold change from baseline due to each individual treatment condition. Consequently, the set of ‘important’ response variables can be identified as those that are statistically significant overall and have a fold change greater than some threshold in at least one treatment relative to the baseline. From this, it can be seen that the conditions identifying the greatest number of ‘important’ response variables are associated with differences in lipid abundance between the genotype Eutrema compared to the abundance in the genotype Columbia. However, these pairwise comparisons make no use of the explanatory structure and it is arguably more appropriate either to obtain a structured summary (see below) or to obtain a single overall measure of biological relevance. For example, Fig. 6b shows two volcano plots, where biological relevance is calculated as the maximal fold increase (and decrease) in any treatment condition compared to the baseline, thus giving a single ranking of ‘importance’ over the response variables.

It is important to emphasise that the (biologically relevant measures of) differences are not necessarily statistically significant, and that the aim here is to produce a ranking considering a measure of *overall* statistical significance coupled with a notion of *overall* biological relevance. For example, lipid X30.0 is considered to be important as it shows both differential expression (a statistically significant one-way analysis) and the largest fold increase in any treatment condition compared to the baseline of no salt stress in Columbia.

#### Marginal assessment

With a complex treatment structure, it will usually be preferable to assess statistical significance for particular treatment terms in the linear model in order to answer specific questions associated with the design of the treatment structure. In a univariate framework, there are three approaches to do this;

1. Assess individual model terms through the appropriately structured ANOVA (or equivalent analysis framework),
2. Incorporate a set of orthogonal contrasts into the treatment structure of the linear model and assess these terms (e.g. through ANOVA),
3. Compare predicted means through pairwise t-tests (also referred to as multiple comparisons or post-hoc tests).

In what follows, we consider extensions of only the first two approaches to the mass univariate framework. There are a multitude of reasons for not considering the third (see, for example, references and discussion in Chapter 8 of [27]).

Since such a marginal assessment will be focussed on the statistical significance of individual model terms, multiplicity corrections should be applied before the predictive model selection. Consequently, for such marginal assessments, the RFM approach is more appropriate.

**Fig. 7:**
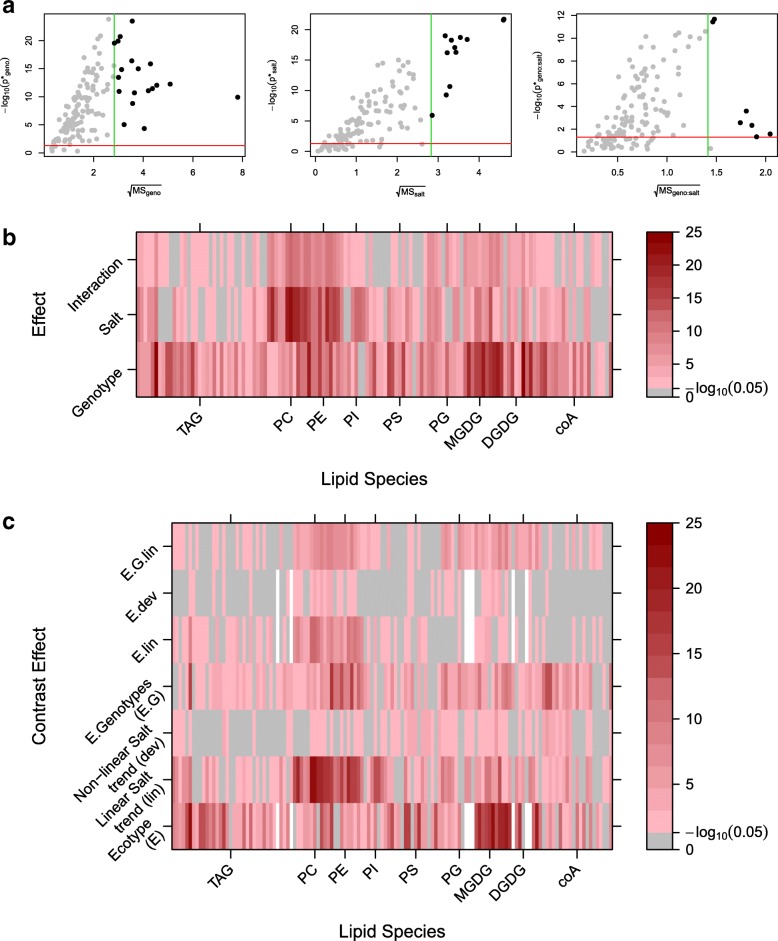
Lipidomics Case Study: Marginal Assessment under the RFM. **a** Generalised volcano plots for each explanatory treatment term within the saturated model, where the term specific *p*-value has been adjusted via the corresponding one-way multiplicity correction under RFM and biological relevance is defined through the treatment mean square. **b** Heatmap of the term specific *p*-values (−*log*_10_(*p*)) adjusted via the corresponding one-way multiplicity correction under RFM. Grey indicates an adjusted *p*-value < 0.05. C) Heatmap of the term specific *p*-values (−*log*_10_(*p*)) for the extended contrast model adjusted via the corresponding one-way multiplicity correction under RFM. Grey indicates a non-significant adjusted *p*-value < 0.05

In contrast to the overall assessments of the previous section, it is now of interest to make multiple assessments for each response variable. For example, consider the subset of lipids found to have two significant main effects but no significant interaction (under RFM). The response variables can be ranked by the significance of the first factor (the variety effect) and also by the significance of the second factor (the salt effect). Unsurprisingly, these give very different rankings of the lipids, both of which are of biological interest. To give meaning to these measures of statistical significance, an associated measure of biological relevance is required. For a two-level factor, this is straightforward and simply results in the standard volcano plot of difference (often presented in fold change) vs. significance. For a factor with more than two levels, a similar approach to the overall assessment could be used. For instance defining relevance as a maximal fold change to a baseline or, where no baseline exists, the maximal fold change between pairs of treatment conditions. However, neither of these approaches maintain the factor definition of a marginal assessment. A natural alternative is to generalise the notion of biological relevance from simple comparative predictions to estimated effects. Explicitly, relevance can be defined by $\sqrt {MS_{T}}$, where *MS*_*T*_ is the treatment mean square for the explanatory term *T*. A larger value of the treatment mean square indicates a greater variation in the mean response of the levels of the treatment factor. Consequently, a biologically relevant threshold, *Thres*^*B*^, can be defined such that only response variables showing a *MS*_*T*_>*Thres*^*B*^ are deemed relevant. Moreover, under the RFM, each treatment mean square is explicitly tested against the null hypothesis that *MS*_*T*_/*MS*_res_=1 at a significance level corrected for multiplicity according to the associated one-way analysis. Since the residual mean square, *MS*_res_, is an estimate of background variation, this hypothesis test provides the corresponding assessment of statistical significance having adjusted for multiplicity. Thus, a generalisation of the volcano plot is obtained (Fig. 7a) for individual explanatory terms.

In addition, the adjusted *p*-values for each treatment term can be obtained for each response variable. This is shown for the lipidomic data in Fig. 7b, where lipids can be identified according to the most significant treatment factor. For instance, the majority of PC lipids are seen to have more significant effects associated with salt stress, while MGDG lipids have more significant effects associated with differences in genotype.

The above assessment of individual factors extends the analysis obtained from ANOVA to the mass univariate framework. In a similar way to a single univariate analysis, a greater level of detail can be obtained by decomposing the explanatory model structure. If each term within the linear model can be parameterised into a set of one degree of freedom terms (or contrasts), than marginal assessment boils down to a set of hypothesis tests that can be directly related to a treatment difference and hence traditional volcano plots can be used. In practice, this will rarely occur but as exemplified in the lipidomics experiment, contrasts can be included to extract a greater level of detail. For example, rather than simply analysing the factor associated with three levels of salt stress, this factor can be decomposed into two parts in order to test for evidence of a linear trend and deviations from such a trend in the quantitative levels of salt stress. Similarly, the factor associated with four different genotypes of *Arabidopsis* can be decomposed into two parts in order to test for a) differences in ecotypes (Eutrema vs. Others) and b) differences in genotypes within the same ecotype (Columbia, Ta-0 and Shadara). These effects are shown in Fig. 7c and again groups of responses showing similar patterns can be extracted.

It is clear that as more complex models are considered this approach of marginal assessment becomes more involved. However, the generalised visualisations extending the interpretation of ANOVA into three dimensions (term, effect size, response) can greatly aid the biological interpretation.

## Discussion

In this paper, two methods have been introduced for incorporating multiplicity corrections in a mass univariate analysis for non-trivial explanatory (treatment) structures. Each approach has its own advantages and the choice between them will depend upon the context of any analysis. The RFM approach has been shown to be conservative in identifying statistically significant response variables, as multiplicity is applied to the one-way test of the saturated model, whereas the MRF approach overcomes this conservativeness through an inflation of the residual degrees of freedom due to a one-way test of the predictive model. Inflating the residual degrees of freedom under MRF may not always be desirable and an alternative would be to partition the treatment sums of squares into two parts, one associated with the one-way treatment structure of the predictive model and the other associated with a ‘lack-of-fit’ term. In this way, a one-way test of the predictive model can be defined whilst maintaining the residual degrees of freedom for which the experiment was designed.

The approach to use will be influenced by the type of analysis used downstream. Overall assessments (a single assessment per response), such as a single ranking, a classification by predictive model or a categorisation of differentially expressed vs. non-differentially expressed response variables naturally fit within the MRF framework. In comparison, marginal assessments (multiple independent assessments per response), such as individual treatment effects or an incorporation of contrast effects are more naturally expressed in the RFM framework.

### Alternative approaches

In this paper, attention has been focussed on controlling the multiplicity of tests of the full explanatory model over the *R* response variables, i.e. at the response variable level within an experiment, through a stage-wise hypothesis testing procedure. Alternatives to this stage-wise paradigm can be found in the literature, but have limitations to the response-level interpretation. Specifically, rather than controlling the FDR at the response-level, an alternative would be to correct for multiplicity over the *R* responses for each of the *p* explanatory terms, i.e. at the explanatory term level within an experiment.

The naïve approach might consider that for *p* explanatory terms over *R* response variables, a total of *R*×*p* tests are required. As such, a global multiplicity correction over this full set of *R*×*p* tests could be applied. However, this approach will be vastly over-conservative. Moreover, for all but the simplest form of correction, interpretation becomes difficult as each of the explanatory terms for a single response variable will be assessed at a different level of significance as, for example, under a Benjamini-Hochberg correction. This prevents the use of a model-based interpretation that combines statistical significance with biological relevance as in the examples above.

In orthogonal balanced designs, it is conceivable that controlling multiplicity for each explanatory term separately is desirable. Specifically, *p* separate multiplicity corrections can be applied for each of the *p* explanatory terms. This then results in the identification of groups of responses that are statistically significant for each separate explanatory term as implemented in [15]. As with the approaches derived in this paper, an additional model selection step can be included to base the multiplicity corrections on the predictive rather than saturated models. Applying these approaches to the simulated data in scenario 1 (Section 4 of the Supplementary Material in Additional file 1), large discrepancies can be seen, particularly in the assessment of significance of the main effects. This is unsurprising, since this Model, Subset, Filter (MSF) approach does not respect the ‘bottom-up’ marginality interpretation of the ANOVA, with main effects potentially omitted without the prior removal of the associated interaction terms. Moreover, the final interpretation becomes limited, as again one cannot analyse the output in a model-based framework as the assessment of individual terms is not consistent within a response variable.

Both the global multiplicity corrections (for all *R*×*p* tests) and separate multiplicity corrections (over all *p* tests for each of *R* terms) are incorporated in the limma package [24] for differential expression analysis of RNAseq and microarray data.

### Extensions

This paper has focussed attention on designed experiments where all terms within a linear model are factors and the associated analysis can be extracted through ANOVA. In practice, many ’omics datasets may be better suited to alternative univariate analysis approaches such as linear mixed models (to account for unbalanced designs), linear and generalized linear models to account for covariates or non-normal responses and hierarchical models to account for known dependence structures. Regardless of the complexity of the individual univariate technique, so long as there is a clear definition of a saturated and predictive model, the principles introduced in this paper will hold.

## Conclusions

Mass univariate approaches provide a valuable complement to multivariate techniques to analyse and interpret ’omics data. In particular, univariate approaches are often well developed for problematic data, for instance, in dealing with missing values, unequal replication, unbalanced designs and autocorrelated error structures, which can be difficult to incorporate in a multivariate setting. Moreover, mass univariate approaches enable statistical significance and biological relevance to be assessed at an individual response variable level in addition to the profile level assessment obtained through a multivariate analysis.

However, as demonstrated in this paper, when analysing ’omics data through a mass univariate approach, a choice of procedures for incorporating multiplicity corrections is available. To gain a deeper understanding of the mechanisms underlying a particular response variable, it is particularly important to assess the full treatment structure of the design rather than the simplified one-way analysis common in the literature. When coupled with an approach to control the multiplicity rate, different analysis approaches give more or less influence to either fitting the predictive model or correcting for multiplicity. Thus, it is important to be aware of the implicit choice that is being made.

It is often the case that as the number of responses increases, ‘simple’ and ‘easy to interpret’ analyses are preferred. This often comes at the cost of statistical rigor, but equally the interpretation of model-based approaches can be cumbersome. However, models generally provide a deeper insight, for instance, defining the classification of responses into subgroups based on the statistical significance of terms in a model or clustering responses based on predictions in different conditions. Two different procedures for obtaining the predictive model whilst also incorporating multiplicity corrections have been introduced and illustrated on data. The approach to use will be driven by the specific aims of the analysis, namely whether a marginal or overall assessment is most appropriate.

Since this choice in methods arises due to the presence of non-trivial (more complex) explanatory treatment structures, this consideration will become increasingly important as the ability to include more complex treatment structures and collect observational covariates becomes more accessible due to the influx of accessible ’omics technologies.

## Additional files

Additional file 1 **Supplementary Material. Section 1** Equating variance ratios. **Section 2** Description of the RMF approach. **Section 3** Simulation study: **Section 3.1** Data generation, **Section 3.2** False discovery rates, **Section 3.3** Results under a Bonferroni correction of the FWER, **Section 3.4** Comparison to limma approaches, **Section 3.5** Comparison of MSF approaches. (PDF 330 kb)

Additional file 2 Lipidomics model assessment. Table containing the overall assessment of statistical significance and the model fitted for each lipid under both RFM and MRF. (PDF 8 kb)

## Abbreviations

ANOVA: Analysis of variance
B-H: Benjamini-Hochberg
FDR: False discovery rate
FWER: Family-wise error rate
MRF: Model, rank, filter
OFDR: Overall false discovery rate
PFER: Per-family error rate
RFM: Rank, model, filter
RCBD: randomized complete block design
