# Supplementary material for: Beyond the one-way ANOVA for ’omics data
Source: BMC Bioinformatics. 2018 Jul 9;19(Suppl 7):199. doi: 10.1186/s12859-018-2173-7 (PMC6069693; doi:10.1186/s12859-018-2173-7)
Supplement: Supplementary file 1 — Supplementary Material. Section 1 Equating variance ratios. Section 2 Description of the RMF approach. Section 3 Simulation study: Section 3.1 Data generation, Section 3.2 False discovery rates, Section 3.3 Results under a Bonferroni correction of the FWER, Section 3.4 Comparison to limma approaches, Section 3.5 Comparison of MSF approaches. (PDF 330 kb) [file 12859_2018_2173_MOESM1_ESM.pdf]

# Supplementary Material: Beyond the one-way ANOVA for 'omics data

Kirsty L. Hassall and Andrew Mead

## 1 Equating variance ratios

Let $VR_{A*B}$ be the one-way variance ratio (F-statistic) associated with the saturated 2 factor model ($t_A \times t_B$ factorial explanatory structure) such that,

$$\begin{aligned} VR_{A*B} &= \frac{N - t_A t_B}{t_A t_B - 1} \times \frac{SS_A + SS_B + SS_{A:B}}{SS_{res}} \\ &= \frac{N - t_A t_B}{t_A t_B - 1} \times \left[ \frac{SS_A + SS_B}{SS_{res}} + \frac{SS_{A:B}}{SS_{res}} \right] \end{aligned}$$

Letting $\gamma := \frac{SS_A + SS_B}{SS_{res}}$ and rearranging,

$$\gamma = VR_{A*B} \times \frac{t_A t_B - 1}{N - t_A t_B} - \frac{SS_{A:B}}{SS_{res}}. \quad (1)$$

Let $VR_{A+B}$ be the one-way variance ratio (F-statistic) associated with the predictive model having dropped the interaction term from the associated linear model such that,

$$\begin{aligned} VR_{A+B} &= \frac{N - (t_A + t_B)}{t_A t_B - 1} \times \frac{SS_A + SS_B}{SS_{res} + SS_{A:B}} \\ &= \frac{N - (t_A + t_B)}{t_A t_B - 1} \times \frac{SS_A + SS_B}{SS_{res}\left(1 + \frac{SS_{A:B}}{SS_{res}}\right)} \\ &= \frac{N - (t_A + t_B)}{t_A t_B - 1} \times \frac{\gamma}{\left(1 + \frac{SS_{A:B}}{SS_{res}}\right)}. \end{aligned}$$

Rearranging for $\gamma$,

$$\gamma = VR_{A+B} \times \frac{t_A t_B - 1}{N - (t_A + t_B)} \times \left(1 + \frac{SS_{A:B}}{SS_{res}}\right). \quad (2)$$

Thus, using (1) and (2), a direct relationship between the two variance ratios obtained under the RFM ($VR_{A*B}$) and MRF ($VR_{A+B}$) can be obtained.

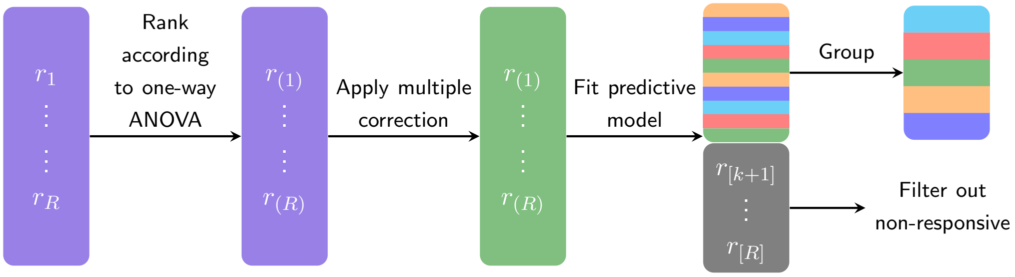


Figure 1: Pictorial representation of the Rank Model Filter (RMF) approach to incorporating multiplicity corrections for non-trivial explanatory structures.

## 2 RMF approach

One way to address the conservativeness of the RFM (rank, filter, model) approach is to move the filtering step until post-model selection. Specifically, this alternative RMF (rank, model, filter) approach takes the following steps,

1. For each of the $n$ response variables, fit the linear model with a single one-way (unstructured) treatment term and calculate the associated one-way ANOVA to obtain an overall test of significance.

2. Rank the responses based on the significance of this overall test and apply a multiplicity correction of choice to this set of $n$ tests.

3. For each response variable, apply a model selection process to the full explanatory structure. This will then define specific explanatory structures for each response, yielding the predictive model for each of the $n$ responses. Note the model selection step will carry through the multiplicity adjustment of step 2, i.e. terms are kept in the predictive model if they satisfy the adjusted significance level.

4. Those responses found to have no significant terms are then filtered out.

This process is depicted in Figure 1

# 3 Simulation study

## 3.1 Data generation

Data were generated according to the specified design matrix for each scenario considered in the simulation study (as listed in the main text). For example, consider the scenario of a $2 \times 2$ factorial design consisting of 500 independent responses with each treatment combination replicated three times. Each response, $r = 1, ...500$, was sampled independently, from $N(\mathbf{X}^{(r)}\beta^{(r)}, \sigma^{2(r)})$, where $\sigma^{2(r)}$ is the background variance for response $r$, $\beta^{(r)}$ is the vector of treatment coefficients and $\mathbf{X}^{(r)}$ is the design matrix. For each $r$, the design matrix is randomly chosen to include;

- no treatment effect: $\mathbf{X}^{(r)} = (1, ...., 1)^T$, $\beta^{(r)} = \mu^{(r)}$
- main effect of treatment 1: $\mathbf{X}^{(r)} = \begin{pmatrix} 1 & 0 \\ 1 & 0 \\ \vdots & \vdots \\ 0 & 1 \\ 0 & 1 \end{pmatrix}$, $\beta^{(r)} = (\tau_1^{(r)}, \tau_2^{(r)})$
- main effect of treatment 2: $\mathbf{X}^{(r)} = \begin{pmatrix} 1 & 0 \\ 1 & 0 \\ \vdots & \vdots \\ 0 & 1 \\ 0 & 1 \end{pmatrix}$, $\beta^{(r)} = (\nu_1^{(r)}, \nu_2^{(r)})$
- main effect of both treatment 1 and treatment 2: $\mathbf{X}^{(r)} = \begin{pmatrix} 1 & 0 & 1 & 0 \\ 1 & 0 & 0 & 1 \\ \vdots & \vdots & \vdots & \vdots \\ 0 & 1 & 1 & 0 \\ 0 & 1 & 0 & 1 \end{pmatrix}$,

  $\beta^{(r)} = (\tau_1^{(r)}, \tau_2^{(r)}, \nu_1^{(r)}, \nu_2^{(r)})$
- main effects and interaction effects of treatment 1 and treatment 2: $\mathbf{X}^{(r)} = \begin{pmatrix} 1 & 0 & 0 & 0 \\ 0 & 1 & 0 & 0 \\ \vdots & \vdots & \vdots & \vdots \\ 0 & 0 & 1 & 0 \\ 0 & 0 & 0 & 1 \end{pmatrix}$, $\beta^{(r)} = (\mu_1^{(r)}, \mu_2^{(r)}, \mu_1^{(r)}, \mu_2^{(r)})$

For each response, each treatment coefficient was sampled from a uniform distribution with specific limits.

The data generation process for the $3 \times 2 \times 4$ factorial design with treatment factors A, B and C followed the same process but incorporated a random choice of 19 design matrices corresponding to the following model definitions,

1. A + B + C + A:B + A:C + B:C + A:B:C
2. A + B + C + A:B + A:C + B:C
3. A + B + C + A:B + A:C
4. A + B + C + A:B + B:C
5. A + B + C + A:C + B:C
6. A + B + C + A:B
7. A + B + C + A:C
8. A + B + C + B:C
9. A + B + C
10. A + B + A:B
11. A + C + A:C
12. B + C + B:C
13. A + B
14. A + C
15. B + C
16. A
17. B
18. C
19. 0

For the final design scenario, the set of responses for each simulation were simulated from a multivariate normal distribution with a block diagonal covariance matrix and a mean vector defined $\mathbf{b} + \mathbf{X}^{(r)}\beta^{(r)}$, where $\mathbf{b}$ is the vector of block effects, assumed to be constant across all responses.

## 3.2 False discovery rates

In addition to the observed error rates summarised in the main paper, for the scenarios applied under a B-H control of the false discovery rate, we summarise here the observed false discovery in Figure 2. In all cases, the majority of the distribution of observed FDRs lie below the 0.05 threshold.

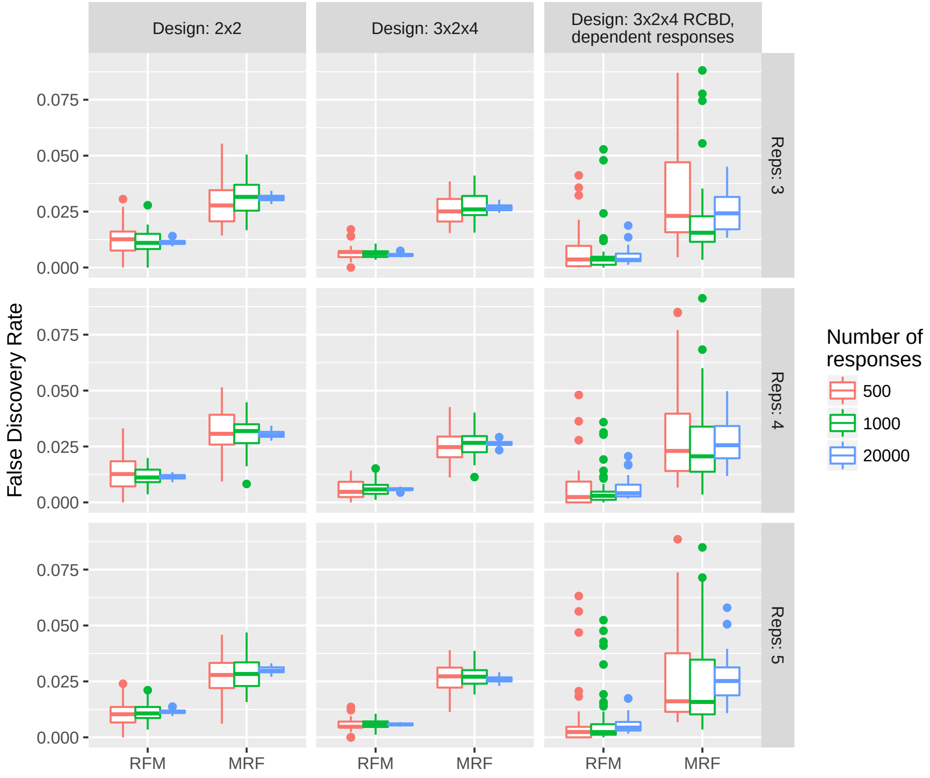


Figure 2: Empirical false discovery rates calculated for each simulated dataset, summarised by each simulation scenario.

### 3.3 Results under a Bonferroni correction of the FWER

Figure 3 shows the rate of type I and type II errors under the RFM and MRF approaches for the simulated datasets under a Bonferroni control of the FWER.

Figure 4 shows the rate of model misspecification under the RFM and MRF approaches for the simulated datasets under a Bonferroni control of the FWER. Fitted models have been classified in one of four ways,

1. Correct specification. The fitted model is in complete agreement with the true model of the data generating process
2. Model over-fitting. The fitted model includes all terms from the true generating process with additional terms.
3. Model under-fitting. The fitted model includes only a subset of terms, and no others, from the true generating process.
4. Model misspecification. The fitted model differs from the true model in a way not encapsulated by under- or over-fitting.

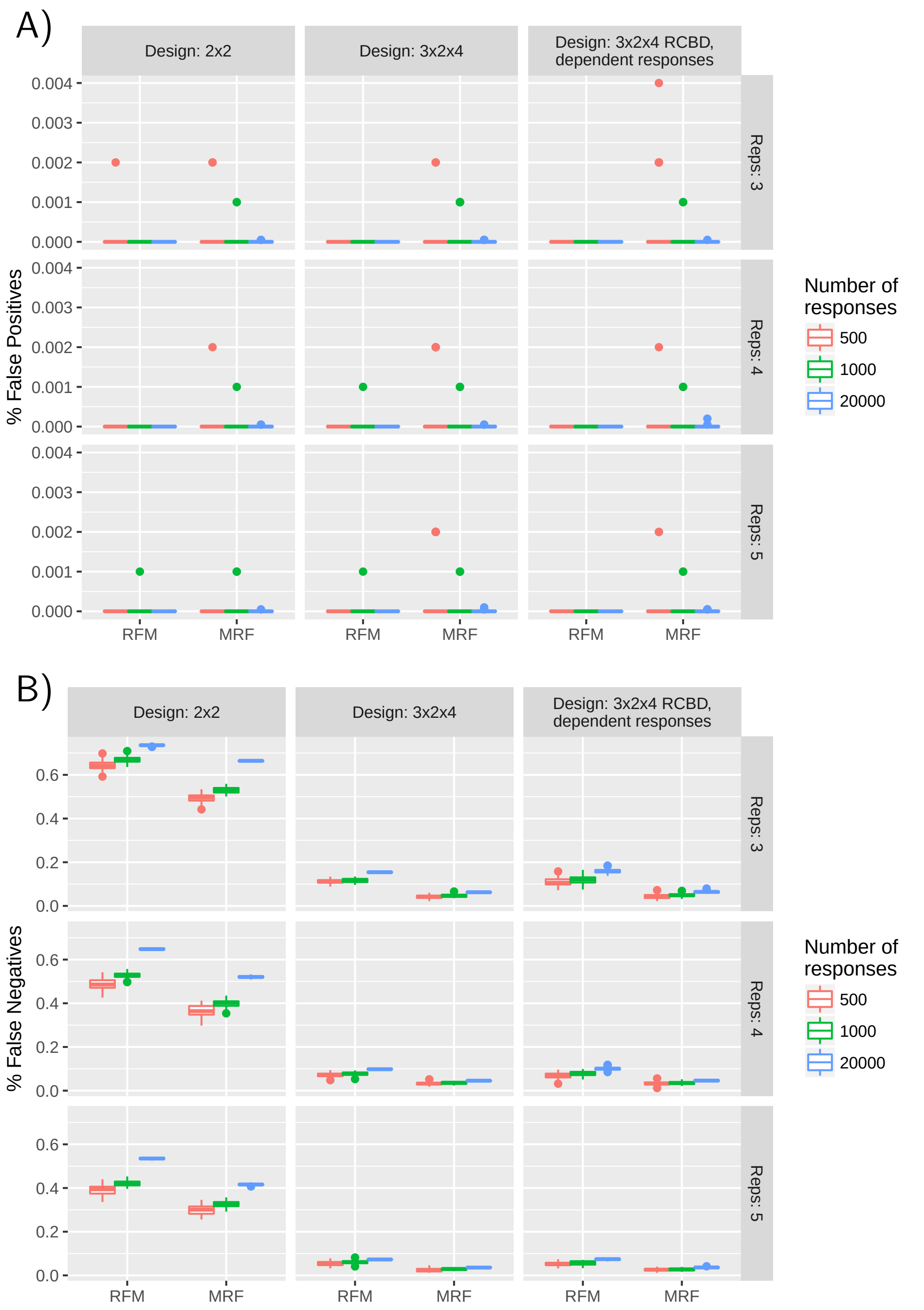


Figure 3: Under a Bonferroni control of the FWER: A) Boxplots showing the distribution of the percentage of responses (within a dataset) falsely identified as having non-constant mean response and B) Boxplots showing the distribution of the percentage of responses (within a dataset) falsely identified as having a constant mean response over all treatment groups.

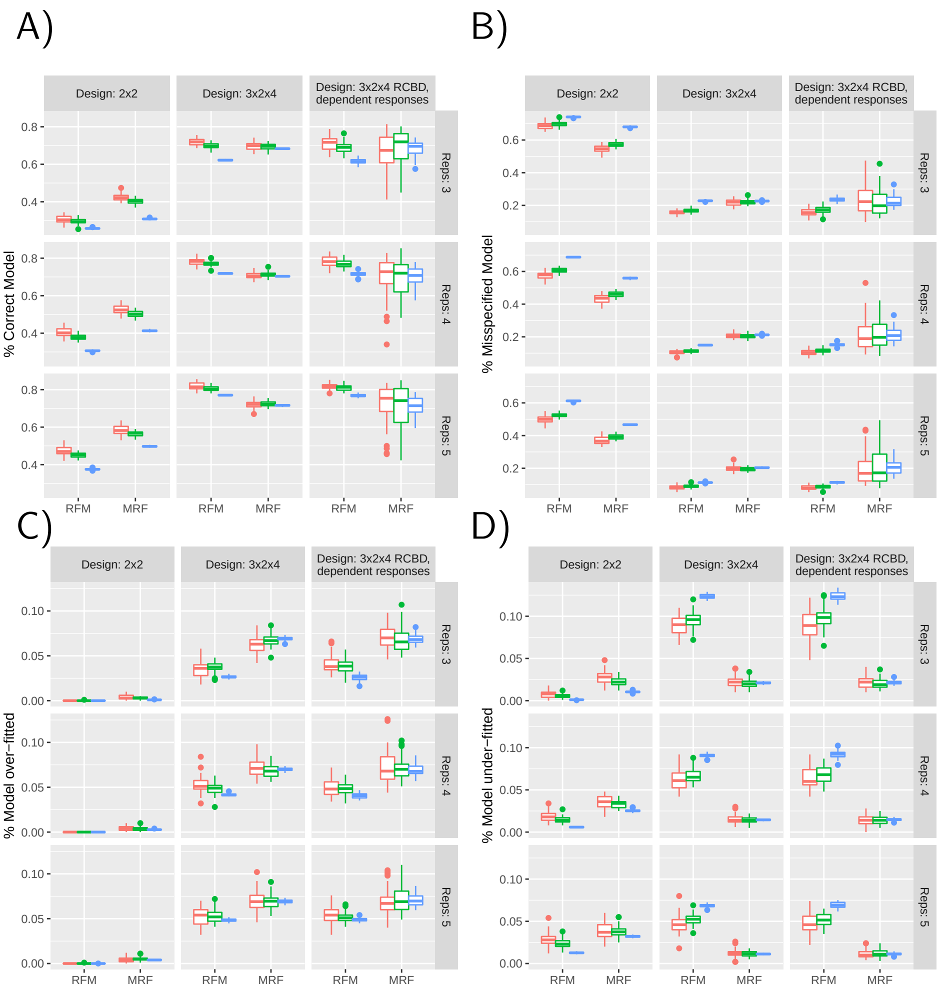


Figure 4: Under a Bonferroni control of the FWER: A) Percentage of responses with the correct model specification identified from each of the RFM and MRF approaches and compared to the true generating process. B) Percentage of responses with a model misspecification identified from each of the RFM and MRF approaches and compared to the true generating process. C) Percentage of responses that have been over-fitted (include all terms from the true generating process among others) under the RFM and MRF approaches. D) Percentage of responses that have been under-fitted (include only a subset of terms, and no others, from the true generating process) under the RFM and MRF. A model misspecification is defined to be a model that differs from the true generating process in a way not captured by over- or under-fitting. Red, green and blue correspond to simulations of 500, 1000 and 20000 responses respectively.

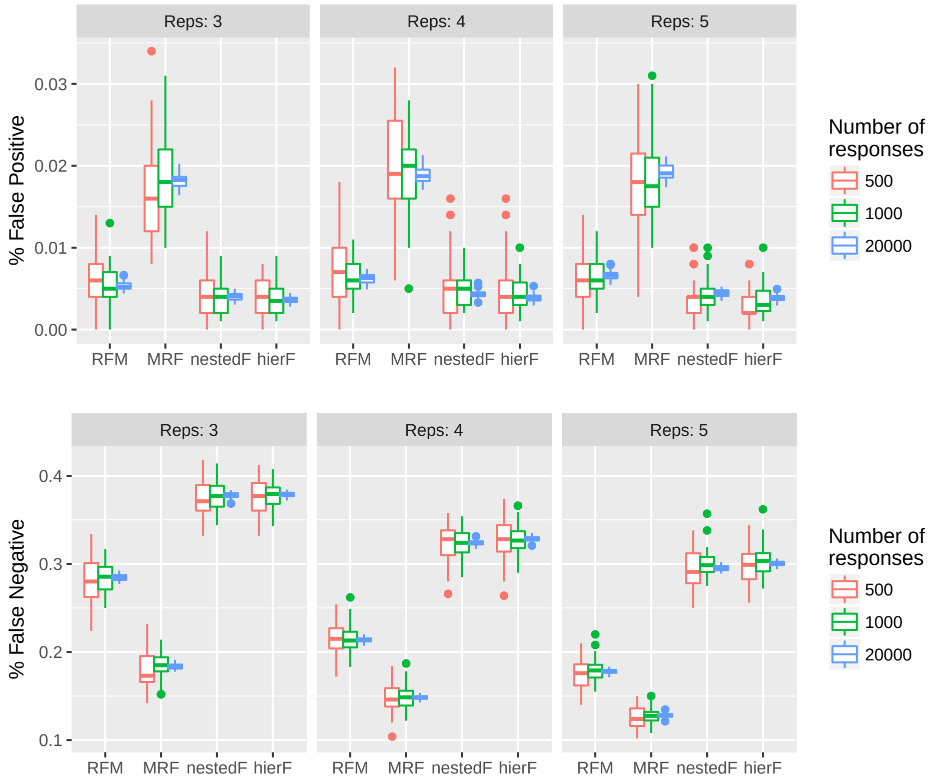


Figure 5: The percentage of type I and type II errors under the RFM, MRF, nestedF and hierF approaches applied to the $2 \times 2$ factorial design structure. Error rates based on the proportion of responses found to be overall differentially expressed.

### 3.4 Comparison to limma approaches

Figure 5 shows the comparison of error rates between the RFM and MRF approaches derived in the main paper and the nested F and hierarchical F approaches implemented in the limma package. It can be seen that the two limma methods perform marginally better than the RFM in terms of the achieved type I error control but it appears to be at the cost of a substantially larger type II error rate.

### 3.5 Comparison to MSF approaches

An alternative approach to controlling multiplicity across an experiment, is to do so for each model term independently. Explicitly, for an explanatory structure with $p$ terms, $p$ separate multiplicity corrections are applied (Figure 6A). Through this approach, one cannot obtain a model-based interpretation as each

term has a potentially different multiplicity adjustment. However, one can investigate each term independently. Comparing the significance of each term to MRF and RFM for a single simulated dataset (Figure 6B), substantial differences can be observed. In particular, MSF detects a greater number of significant effects.

Extensions to incorporate a model selection step have been considered. For example, a model selection step can be incorporated by first subsetting the responses according to the predictive model (including only terms that are significant at the 5% level) and for each subset, independently applying a separate multiplicity correction to each of the model terms as depicted in Figure 7A. Applying these approaches to a single representative simulated dataset (Figure 7B), we obtain a more conservative implementation of the MRF approach.

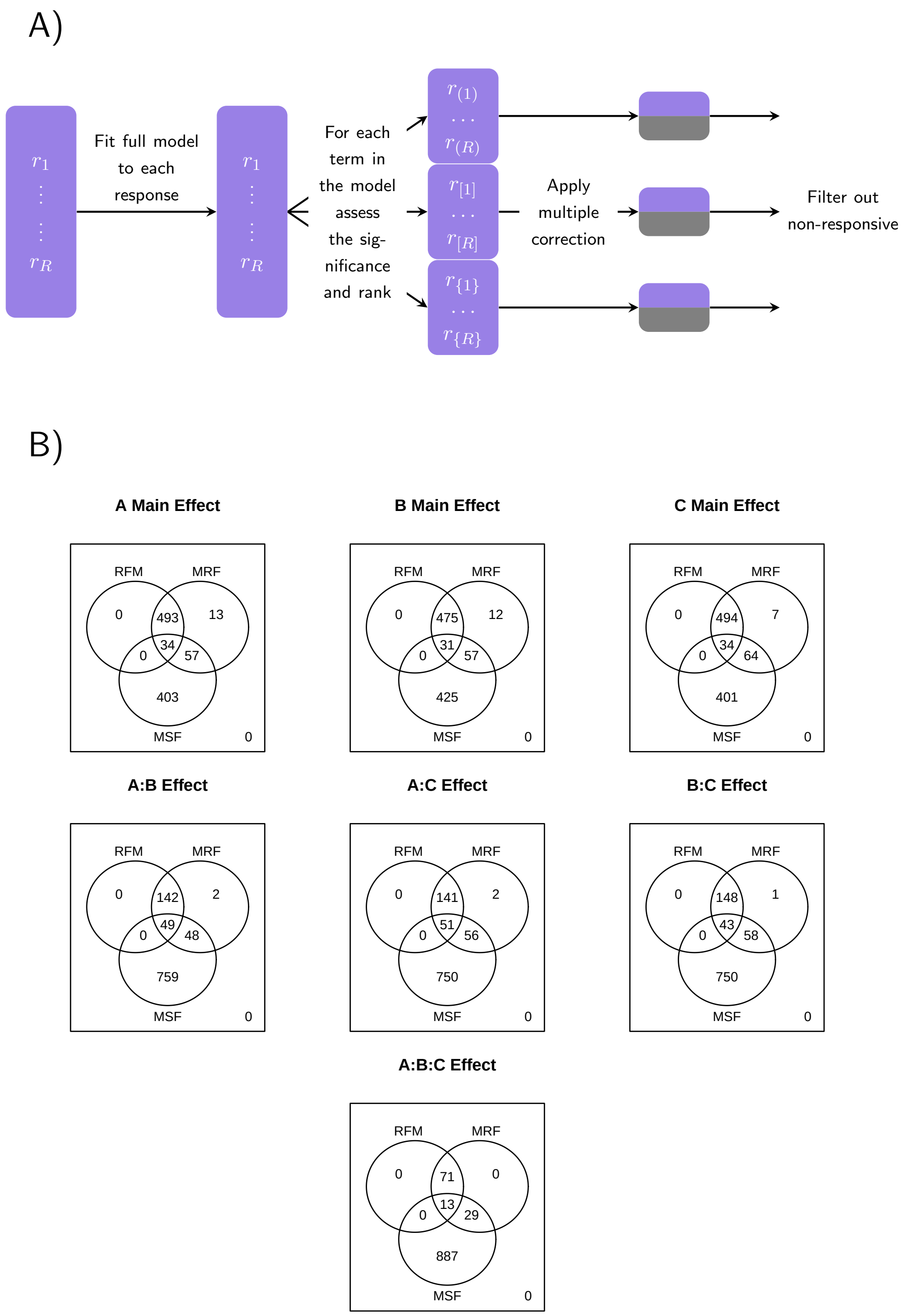


Figure 6: A) Pictorial representation of the Model, Subset, Filter (MSF) approach to incorporating multiplicity corrections for non-trivial explanatory structures. For each term in the explanatory model, a separate multiplicity correction is applied. B) A comparison of the significant terms found under MSF, RFM and MRF.

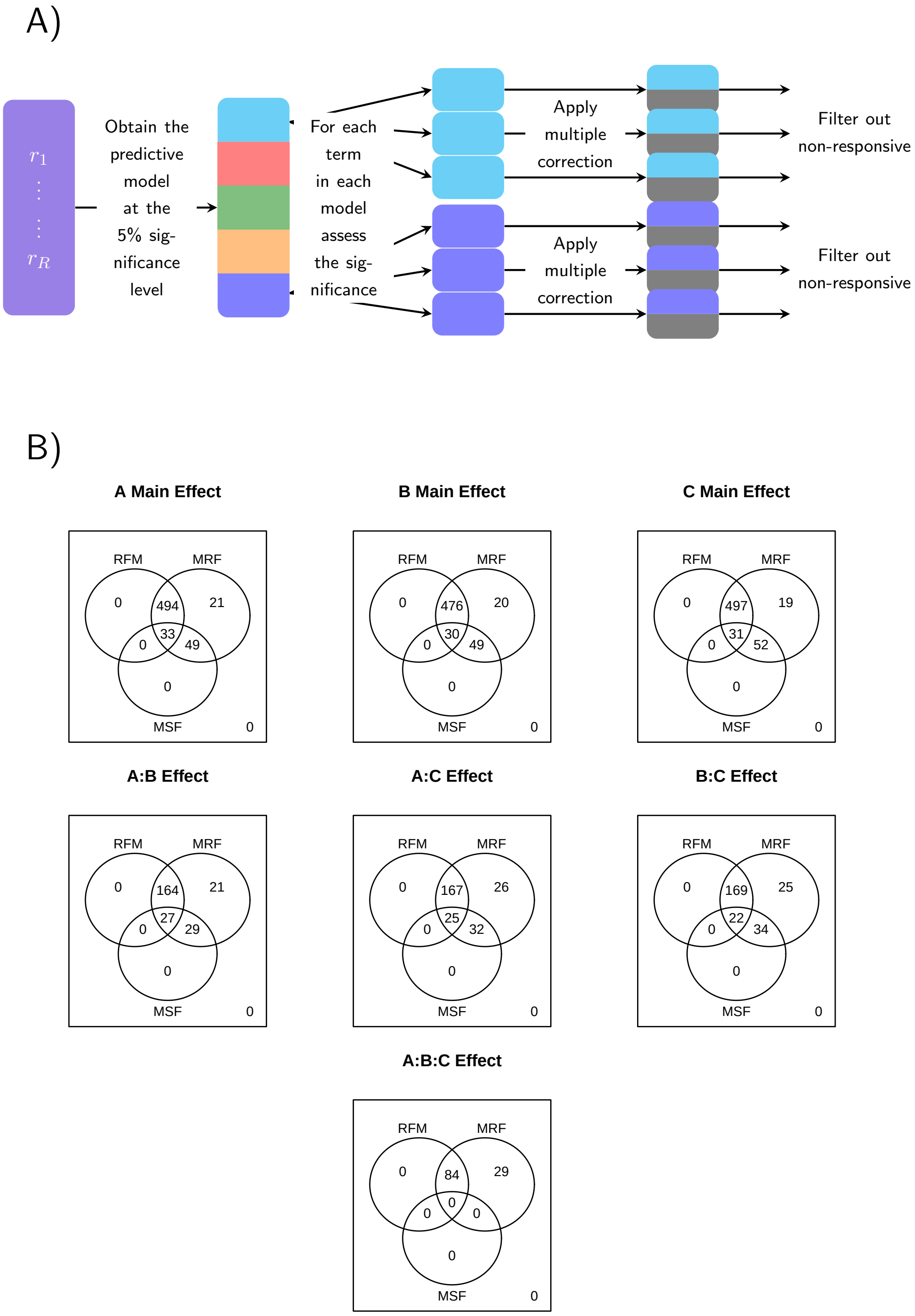


Figure 7: A) Pictorial representation of the extended Model, Subset, Filter (MSF) approach to incorporating multiplicity corrections for non-trivial explanatory structures. An additional model selection step is incorporated by first subsetting the responses according to the predictive model (including only terms that are significant at the 5% level) and for each subset independently applying a separate multiplicity correction to each of the model terms. B) A comparison of the significant terms found under the extended MSF, RFM and MRF.
